# Piezo-catalytic immunotherapy: mechanisms and feasibility in cancer treatment

**DOI:** 10.7150/thno.114676

**Published:** 2025-05-09

**Authors:** Zhiguang Chen, Liang Sang, Donglin Bian, Yanjun Liu, Zhiqun Bai

**Affiliations:** Department of Ultrasound, The First Hospital of China Medical University, No. 155, Nanjing North Street, Heping District, Shenyang, 110001, Liaoning Province, China.

**Keywords:** Piezoelectric effect, Immunotherapy, Sonodynamic therapy, Sono-piezo dynamic therapy, Tumor immune microenvironment

## Abstract

Over the past decade, immunotherapy has revolutionized the clinical management of numerous cancer types. However, only a subset of patients derives long-term durable tumor control from it. Intriguingly, several emerging therapeutic strategies harnessing reactive oxygen species (ROS)-mediated immunogenic cell death (ICD) hold promise for enabling precision immunotherapy in cancer, with sonodynamic therapy emerging as a notable example. The utilization of piezoelectric materials as sonosensitizers can effectively enhance ROS production, thereby augmenting the efficacy of ICD-induced immunotherapy. Additionally, electrical stimulation generated by the piezoelectric effect can further induce immune response activation and necroptosis, achieving a synergistic effect in piezo-catalytic immunotherapy. Herein, we will systematically review the pathways and potential mechanisms underlying piezo-catalytic immunotherapy, offering novel insights for the exploration of cancer immunotherapy strategies.

## Introduction

Cancer immunotherapy, which leverages the body's immune system to recognize and eliminate cancer cells, has emerged as a promising treatment modality for various cancer types 1. This approach encompasses immune checkpoint blockade (ICB) 2, therapeutic vaccines 3, and adoptive cell therapy 4, among others. Over the past decade, immunotherapy has revolutionized the clinical management of numerous cancer types. However, due to significant interindividual variability in response, only a subset of patients achieves long-term durable tumor control 5. Furthermore, immunotherapy can induce adverse events, potentially damaging multiple organ systems and even leading to death in some patients 6,7. Therefore, achieving tumor-targeted immunotherapy is crucial for efficient and low-toxicity cancer treatment 8. Studies have demonstrated that combining immunotherapy with other treatment modalities, such as chemotherapy, radiotherapy, and targeted therapy, can enhance tumor treatment efficacy 9-12.

Intriguingly, emerging therapeutic strategies leveraging reactive oxygen species (ROS)-mediated immunogenic cell death (ICD) have the potential to enable precision immunotherapy for cancer 13. These strategies include sonodynamic therapy (SDT) 14, photodynamic therapy (PDT) 15, and radio-dynamic therapy (RDT) 16. Compared to PDT and RDT, SDT-based immunotherapy offers advantages of low side effects and deep tissue penetration, thus holding greater promise for clinical translation 13. The exact mechanism of SDT remains unclear, but it is believed that the ultrasonic cavitation effect plays a pivotal role. In the presence of sonosensitizers, stable cavitation during the ultrasonic process generates mechanical shearing, micro-jetting, and acoustic microstreaming, lowering the energy threshold required for cell membrane damage. Inertial cavitation triggers sonoluminescence, sonothermal, and sonomechanical effects, resulting in sufficient ROS production to kill cells 17,18. Consequently, SDT can effectively induce ICD and activate antitumor immune responses through ROS-mediated apoptotic pathways in cancer cells 19. However, traditional sonosensitizers face challenges due to low bioavailability, and SDT-induced antitumor immunity is limited by the hypoxic tumor microenvironment (TME) and insufficient ROS production 20. Recent advances in ROS-based therapeutic strategies have been propelled by both endogenous response modulation and exogenous stimulation techniques. For endogenous potentiation, electron hybridization-mediated chemical dynamics (e.g., transition metal-catalyzed Fenton-like reactions) can amplify intratumoral oxidative stress through rational nanoplatform design 21,22. Externally triggered approaches offer spatiotemporal control, including: (i) magneto-electrodynamic systems that convert alternating magnetic fields into localized ROS bursts via nano-transducers 23, (ii) energy-band-engineered photodynamic agents that overcome hypoxia limitations by modulating electron-hole separation 24, and (iii) multifield-coupled piezoelectric catalysis integrating mechanical, thermal, and electrical stimuli for deep-tissue therapy 25. These paradigm-shifting strategies collectively expand the frontiers of nanocatalytic medicine by addressing tumor microenvironment constraints while enabling precision therapeutics.

Excitingly, piezoelectric materials have been utilized as sonosensitizers, generating more ROS upon ultrasonic excitation and demonstrating excellent therapeutic efficacy in cancer treatment. This method has been termed sono-piezo dynamic therapy (SPDT) 26-28. In contrast to conventional SDT, which primarily depends on sonosensitizers (e.g., porphyrins or TiO_2_) to generate ROS via ultrasonic cavitation, SPDT introduces a paradigm shift by leveraging the intrinsic piezoelectric properties of materials to achieve dual mechanical-electrical stimulation. Under ultrasound excitation (1-3 MHz), piezoelectric materials undergo electron-hole pair separation, creating a built-in electric field with surface charges that directly drive redox reactions (e.g., H_2_O → •OH, O_2_ → •O_2_^⁻^) at potentials exceeding those of traditional SDT. Critically, engineered piezoelectric heterojunctions enhance charge separation efficiency, enabling sustained ROS production even under hypoxic condition—a limitation that severely restricts SDT efficacy 29. Currently, piezoelectric materials are being explored in cancer immunotherapy, where ultrasonic excitation generates ROS and piezoelectric effects to induce immune response activation and necroptosis, thereby achieving a synergistic effect in tumor piezo-catalytic immunotherapy 30. In this study, we aim to clarify the concept of piezo-catalytic immunotherapy, focusing on elucidating the pathways and potential mechanisms involved. Importantly, we will also discuss the feasibility, advantages, and disadvantages of using piezoelectric materials as sonosensitizers in cancer immunotherapy. Our findings provide new insights for exploring cancer immunotherapy strategies.

### Tumor immune microenvironment (TIM)

**Figure 1:**
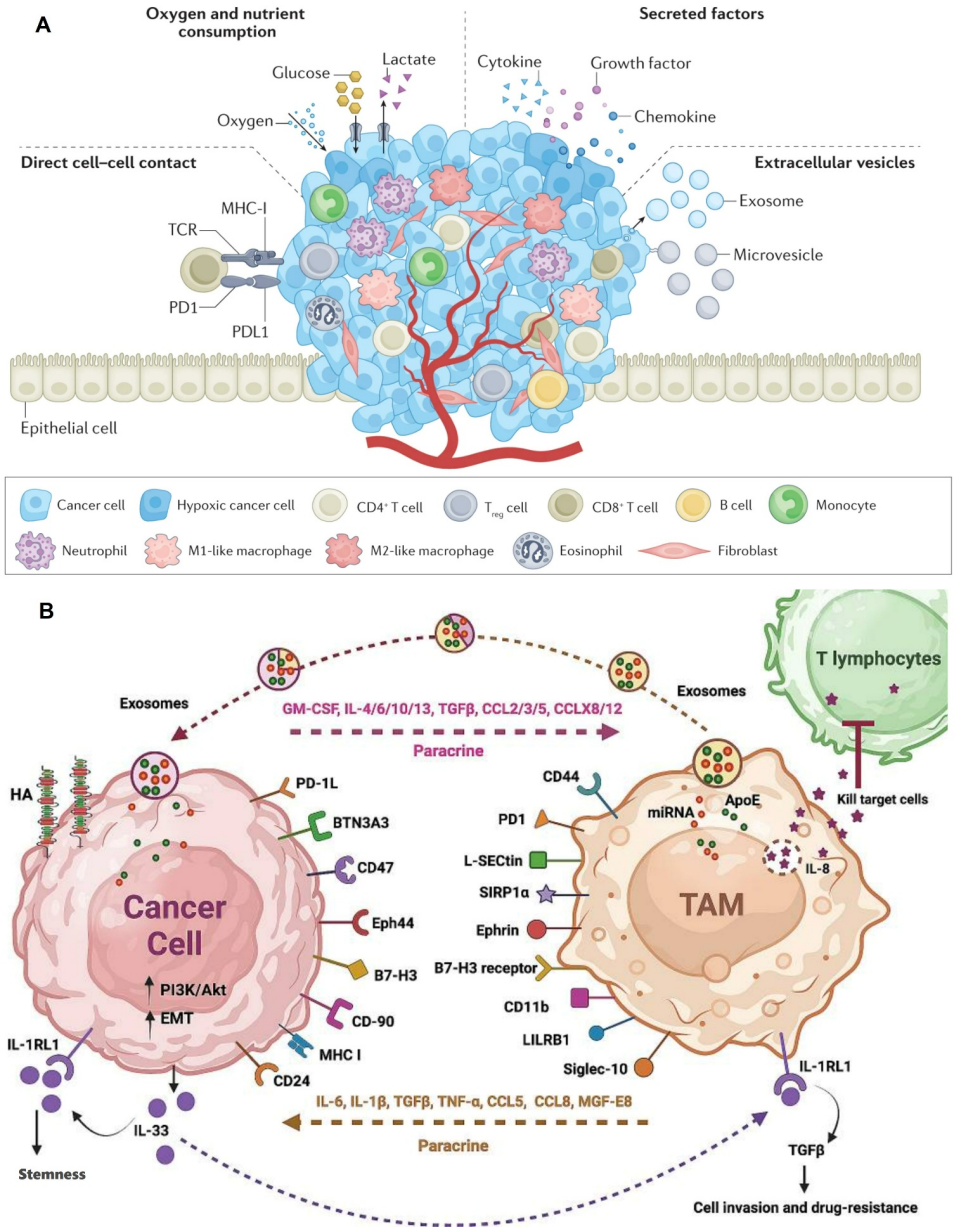
** Tumor immune microenvironment. A)** The communication mechanism between cancer cells and immune cells. Adapted with permission from 33, copyright 2023 Springer Nature Limited; **B)** Interference between tumor cells and TAMs. Adapted with permission from 37, copyright 2024 The Author(s).

The TIM constitutes an intricate network comprising diverse immune cells, immune molecules, and non-immune cellular entities within the tumor tissue and its adjacent milieu. These constituents, through their intricate interactions and regulatory mechanisms, collectively modulate tumor growth, invasion, and metastasis 31,32. Among these components, immune cells emerge as the most prevalent and complex participants within tumor tissue, exhibiting dual functionalities in cancer initiation and progression, exerting both antitumor and protumor effects 33. This duality is primarily contingent on the dynamic crosstalk between cancer cells and immune cells, with cancer cells communicating and nurturing adjacent or distant immune cells via the secretion of extracellular vesicles, histones, cytokines, chemokines, growth factors, and proteases **(Figure 1A).**

Immune cells encompass T cells, B cells, macrophages, and dendritic cells (DCs). Antitumor immune cells primarily consist of effector T cells, specifically CD4^+^ T cells and CD8^+^ T cells, DCs, and M1-polarized macrophages 34. The infiltration level of T cells is regarded as a pivotal determinant for the prognosis of cancer patients. In the natural TME, T cells serve as the primary effector cells of antitumor immune responses. CD4^+^ T cells can augment the proliferation of cytotoxic T lymphocytes and enhance antigen presentation by DCs, whereas DCs produce TNF-α, IL-6, IL-8, and IL-12 to participate in antitumor immunity 35,36. Protumor immune cells predominantly include regulatory T cells (Tregs), M2-polarized macrophages, and N2-polarized neutrophils 31. Notably, M2 macrophages dominate within tumor-associated macrophages (TAMs), inhibiting antitumor immune responses through the secretion of immunosuppressive cytokines such as TGF-β, IL-4, IL-10, among others 37. Consequently, current TIM-targeted therapies primarily concentrate on inducing TAM depletion, inhibiting TAM recruitment, and polarizing M2 macrophages towards the M1 phenotype **(Figure 1B)** 38,39**.**

However, during cancer progression, tumor cells incessantly absorb nutrients to sustain their rapid proliferation. Any alterations in the metabolic process can regulate the survival and function of immune cells, ultimately facilitating immune evasion and tumor progression 40. Attributable to the active glycolysis of tumor cells, the interstitial glucose content within tumors diminishes, and such low glucose levels can impair T-cell functionality 41. Concurrently, the extensive glucose metabolism in tumor cells results in lactate accumulation, with high lactate concentrations impeding CD8^+^ T-cell proliferation and cytokine secretion, thereby influencing the inactivation of tumor-infiltrating lymphocytes (TILs) 42. Furthermore, the TME is abundant in lipids, encompassing fatty acids, lipoproteins, and cholesterol. Lipid accumulation in DCs induces endoplasmic reticulum (ER) stress, diminishing antigen presentation capabilities. ER stress induced by cholesterol accumulation leads to T-cell exhaustion. Enhanced lipid intake and peroxidation result in heightened oxidative stress, culminating in CD8^+^ T-cell dysfunction and ferroptosis 43,44. Therefore, targeted interventions directed at any of these processes possess the potential to achieve immunotherapy for cancer.

### Sonodynamic therapy for TIM

**Figure 2:**
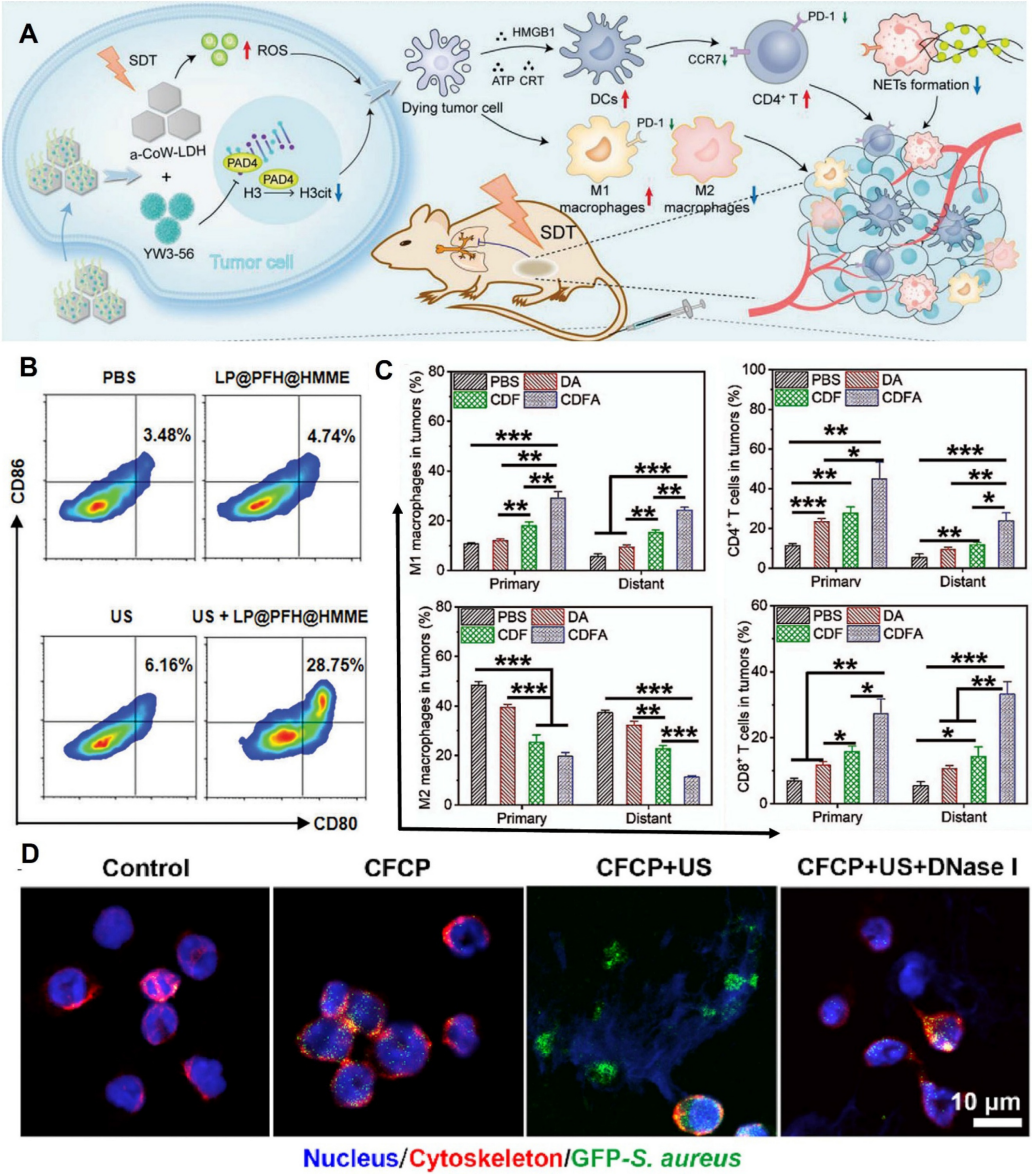
** Sonodynamic therapy for TIM. A)** Schematic illustration of the preparation of a-LDH@356-PEG nanosheets and their application in synergistic SDT/immunotherapy. Adapted with permission from 46, copyright 2024 The Author(s). **B)** A typical flow cytometry assay of mature BMDCs (CD80+CD86+). Adapted with permission from 48, copyright 2024 The Author(s). **C)** Quantitation analysis of M1 TAM(CD45^+^CD11b^+^CD80^+^F4/80+), M2 TAM(CD45^+^CD11b^+^CD206^+^F4/80^+^), CD4^+^ T cells (CD45^+^CD3e^+^CD4^+^), CD8^+^ T cells (CD45^+^CD3e^+^CD8^+^). Adapted with permission from 49, copyright 2023 Elsevier Ltd. **D)** CLSM images after co-culturing neutrophils from different DCM co-culture groups with fluorescent bacteria. Green for GFP-S. aureus, red for the neutrophil cytoskeleton, and blue for the nucleus. Adapted with permission from 54, copyright 2024 The Author(s).

The three essential elements of SDT are ultrasound stimulation, sonosensitizers, and oxygen. The development of sonosensitizers has evolved from the earliest porphyrins and their derivatives to today's metal-organic framework sonosensitizers and novel piezoelectric sonosensitizers, undergoing a process from simplicity to complexity and from singularity to diversity 45. As research deepens, scholars 46 have discovered that SDT can trigger systemic immune responses in the body and promote the treatment of metastatic tumors. For example, amorphous CoW-layered double hydroxide loaded with peptidylarginine deiminase 4 (PAD4) generates a large amount of ROS under ultrasound, inducing ICD while inhibiting the increase in citrullinated histone H3 (H3cit) and the release of neutrophil extracellular traps (NETs). This method upregulates the antitumor response while inhibiting immune escape **(Figure 2A)** and is defined as sono-immunotherapy 47.

Effective immunotherapy can be achieved by enhancing ICB through SDT. Nanoparticles loaded with anti-PD-L1 antibodies and containing sonosensitizers are constructed to generate a certain amount of ROS under ultrasound excitation, triggering ICD to sensitize the immune efficacy of ICB induced by anti-PD-L1 48-50. However, the mechanisms of action of SDT vary. Chen et al. 48 found that ICD induced by SDT may further trigger antitumor immune activation by promoting DC maturation **(Figure 2B)**. In contrast, Ya *et al*. 49 found that amplified ICD combined with ICB increased the infiltration of cytotoxic T lymphocytes and natural killer (NK) cells, polarized M2 macrophages into M1 macrophages, and reduced regulatory T cells **(Figure 2C)**.

SDT-mediated ICD activates the stimulator of interferon genes (STING) to amplify the immune stimulation of tumor-infiltrating myeloid cells. Based on STING agonists such as MSA-2 51 and Mn^2+^ 52, nanomedicine delivery systems are constructed to generate ROS under ultrasound excitation, accurately damaging the cell nucleus while activating the cGAS-STING pathway to induce innate immune responses 53. Alternatively, interference with iron ion metabolism increases the release of bacterial-associated double-stranded DNA (dsDNA), which promotes DC maturation by activating STING, thereby amplifying the immune stimulation of neutrophils **(Figure 2D)** 54.

By targeting the glycolysis process in the TME, SDT significantly inhibits cancer cell glucose uptake and lactate excretion while inducing cancer cell apoptosis and ICD, achieving ultrasound-triggered immune activation 55,56. In addition, SDT synergizes with autophagy 57, oncolytic pyroptosis 58,59, and ferroptosis 56 to maximize tumor immunogenicity and reverse the immunosuppressive TME, ultimately triggering a powerful and durable systemic antitumor immune response.

However, traditional sensitizers have limited ability to generate ROS after ultrasound excitation, resulting in suboptimal efficacy of sono-immunotherapy. Therefore, it is crucial to explore potential sonosensitizers with higher ultrasound responsiveness. Promising candidate materials that have been explored include piezoelectric materials, defective semiconductors, hybrid assembly polymers with narrow band gaps, and novel sono-catalysts with heterojunctions 60.

### The mechanism of SPDT

Traditional SDT relies on inertial cavitation to generate transient ROS bursts, which are often insufficient to induce robust ICD in hypoxic or immunosuppressive TMEs. In contrast, SPDT employs piezoelectric materials to achieve dual energy conversion. The dissociated electrons and holes migrate to opposite surfaces, leading to the production of a certain quantity of ROS through redox reactions. This process facilitates sonodynamic therapy for tumors. Furthermore, the charges induced on the surface of piezoelectric materials can directly impact cancer cells, altering the cell membrane potential, causing depolarization of both the mitochondrial membrane and the plasma membrane, and ultimately enhancing the effectiveness of sonodynamic therapy sensitized by the piezoelectric effect 28. Consequently, piezoelectric materials are regarded as a promising class of sonosensitizers. Currently, inorganic piezoelectric materials are extensively studied due to their superior piezoelectric properties. However, their clinical application is hindered by factors such as high polarization temperatures, elevated preparation costs, poor stability, and potential toxicity. Recent advances in piezoelectric nanocomposites (e.g., 0.7BiFeO_3_-0.3BaTiO_3_ heterojunctions) demonstrate dual capabilities of ROS generation and PD-L1 downregulation, thereby enhancing checkpoint blockade efficacy 61. Their team designed a tumor-targeted BTO-Pd-MnO_2_-HA piezoelectric nanocomposite. This system utilizes Schottky junctions to promote electron-hole separation, significantly boosting ROS production. Additionally, the TME triggers the degradation of MnO_2_, releasing Mn²⁺ ions that catalyze H_2_O_2_ decomposition via Fenton-like reactions to generate hydroxyl radicals (•OH). Concurrently, the nanocomposite depletes glutathione (GSH) and releases oxygen, synergistically enhancing SDT and chemodynamic therapy (CDT). This innovation expands the application of piezoelectric nanomaterials in biomedicine for precise regulation of the tumor microenvironment 62.

In previous research, a comprehensive review of the current status of piezoelectric materials in SDT applications was conducted. Additionally, the concept of SPDT was proposed, and the process of ROS generation by ultrasound-excited piezoelectric materials was elaborated. Specifically, ultrasound stimulates piezoelectric materials, causing the negative voltage at the conduction band (CB) edge to slightly exceed the O_2_/•O_2_^⁻^ redox potential (-0.33 V), thereby energetically favoring the O_2_/•O_2_^⁻^ redox reaction on the CB. Simultaneously, the positive voltage at the valence band (VB) edge is considerably higher than the H_2_O/•OH redox potential, facilitating the direct transfer of hole charges from the VB to water molecules to generate •OH. By constructing piezoelectric heterojunctions, semiconductor materials with lower energy levels are integrated with piezoelectric materials. Following ultrasound excitation, the electron-hole pairs within the piezoelectric materials transfer to the semiconductors with lower energy levels, preventing the rapid recombination of electron-hole pairs within the piezoelectric materials. This process increases ROS production and further promotes tumor cell death. For a detailed introduction, please refer to the recently published article 63. Piezoelectric materials currently applied in immunotherapy are summarized in Table 1.

### Piezo-catalyzed immunotherapy

As early as 2023, Shi and their team proposed the concept of Piezocatalytic Medicine (PCM), which refers to the application of piezocatalytic technology in the medical field. This encompasses the medical applications of piezocatalytic materials in tumor therapy, antibacterial treatments, organic degradation, tissue repair and regeneration, as well as biosensing 64. In recent years, researchers have employed piezoelectric materials for cancer immunotherapy. Under ultrasound excitation, the generated ROS trigger ICD, thereby enhancing the efficacy of immunotherapy for cancer cells by remodeling the immune status of the tumor microenvironment. This approach is termed piezocatalytic immunotherapy 30. While the underlying mechanisms remain incompletely understood, current research indicates that piezoelectric stimulation can modulate immune cells within the tumor immune microenvironment and regulate metabolic components, ultimately reshaping the tumor microenvironment to achieve immunotherapy for tumors.

### Piezoelectric modulation of macrophage in TIM

The immune infiltration within the TME comprises macrophages, microglia, myeloid-derived suppressor cells, lymphocytes, NK cells, and neutrophils. The predominant populations are macrophages and microglia, collectively termed TAMs. The dynamic interplay between these components and glioma cells modulates tumor cell growth, proliferation, invasion, and survival 65,66. Single-cell RNA sequencing has elucidated the evolution of the immune landscape throughout the progression of brain gliomas, revealing that early TME are primarily inhabited by microglia, whereas late-stage environments exhibit substantial infiltration of immunosuppressive macrophages and neutrophils 67. Modulating the phenotype of TAMs can thus reshape the TIM, enabling immune sensitization therapy for tumors.

Montorsi *et al*. 68 treated microglia with piezoelectric polystyrene nanoparticles (PNPs) followed by ultrasound stimulation. They observed that microglia treated with CM-PNPs+US (conditioned medium-coated piezoelectric polystyrene nanoparticles combined with ultrasound stimulation) exhibited augmented inflammatory responses, evidenced by upregulated CD86 marker expression and increased ROS production. Glioblastoma cells cocultured with these stimulated microglia displayed reduced survival and proliferation rates, suggesting anticancer phenotype polarization. This study introduces a novel strategy of utilizing piezoelectric nanomaterials in conjunction with ultrasound stimulation to regulate microglia behavior for antitumor purposes, presenting a potential new approach for glioma immunotherapy. However, this study has limitations. Specifically, the underlying mechanisms through which piezoelectric stimulation enhances the inflammatory response of microglia require further exploration. Additionally, the long-term effects of this treatment on microglia function and cancer progression remain uncertain.

**Figure 3:**
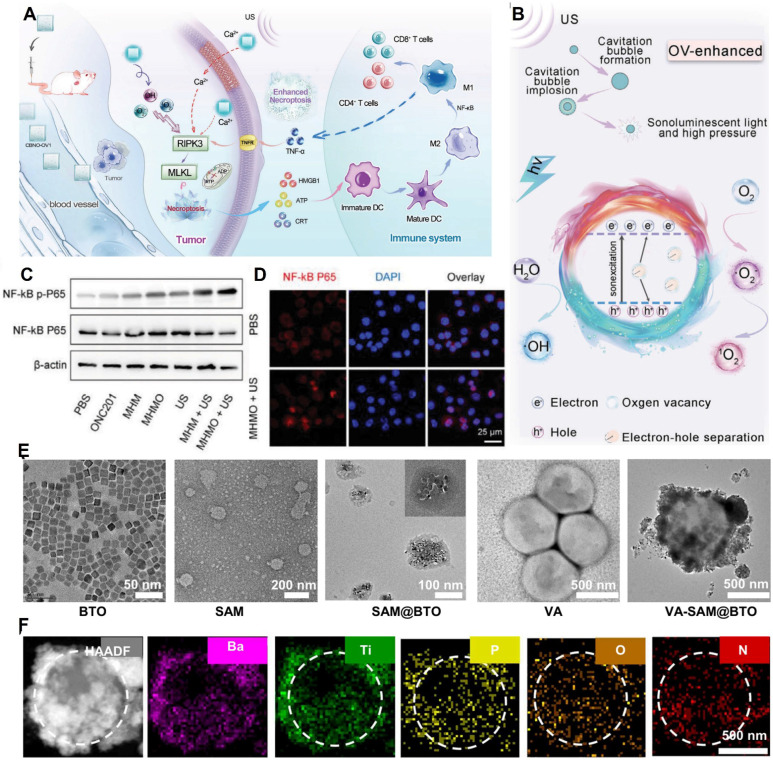
** The piezoelectric effect causes an increase in polarization of M1 macrophages in TIM. A)** Schematic diagram of anti-tumor process inducing necroptosis in tumor cells, activating immune response and further amplifying necroptosis through immunotherapy. Adapted with permission from^69^, copyright 2023 Wiley-VCH. **B)** The schematic diagram of proposed mechanism of CBNO-OV1 NSs for OV-enhanced piezoelectric catalytic therapy. Adapted with permission from 69, copyright 2023 Wiley-VCH. **C)** Western blot analysis demonstrating the activation status of the NF-κB pathway in different treatment groups. Adapted with permission from 70, copyright 2024 The Author(s). **D)** Immunofluorescence showing P65 nuclear translocation following MHMO + US treatment. The scale bar represents 25 μm. Adapted with permission from 70, copyright 2024 The Author(s).** E)** SEM characterization of micro robots constructed based on VA. Adapted with permission from 73, copyright 2024 The Author(s). **F)** high- angle annular dark- field (hAAdF) and mapping im ages of VA- SAM@BTOb. Adapted with permission from 73, copyright 2024 The Author(s).

Du *et al*. 69 fabricated ultrathin CaBi_2_Nb_2_O_9_ nanosheets with tunable oxygen vacancies (CBNO-OV1). Oxygen vacancies enhance electron-hole separation efficiency by inhibiting electron-hole recombination, thereby substantially boosting ROS production. By adjusting the proportion of oxygen vacancies, they determined that the band gaps of CBNO, CBNO-OV_0.5_, CBNO-OV_1_, and CBNO-OV_2_ were 3.23 eV, 3.18 eV, 3.16 eV, and 3.13 eV, respectively. The CB of CBNO, CBNO-OV_0.5_, CBNO-OV_1_, and CBNO-OV_2_ relative to the normal hydrogen electrode was estimated at -0.348 eV, -0.388 eV, -0.438 eV, and -0.508 eV, respectively. Hence, a higher proportion of oxygen vacancies results in higher ROS generation efficiency. Furthermore, the efficient piezoelectric effect increases cell membrane permeability, leading to increased Ca^2+^ influx and necrotic apoptosis of cells. **(Figure 3A and Figure 3B)**.

Yang *et al*. 70 designed piezoelectric Mg^2+^-doped hydroxyapatite (Mg-HAP) nanoparticles coated with a mesoporous silica layer and loaded with ONC201 as an agonist to specifically target the death receptor DR5 on tumor cells. They developed the Mg-HAP@MS/ONC201 nanoparticle (MHMO NP) system with a band gap of 5.3 eV, a VB of +3.55 eV, and a CB estimated at -1.75 eV, which is substantially higher than the redox potentials of H_2_O and O_2_. This study revealed for the first time that MHMO can promote significant ROS release under piezoelectric catalysis. Simultaneously, Ca^2+^ release synergizes with ROS to stimulate necrotic apoptosis of tumor cells, overcoming apoptosis resistance. **(Figure 3C and Figure 3D)**.

Sun *et al*. 71 prepared one-dimensional oxygen-deficient BaTiO_3_ nanorod arrays (BaTiO_3-x_) on Ti to enhance sonocatalytic efficiency. L-Arginine (LA) was covalently immobilized on the surface of BaTiO_3-x_ (BaTiO_3-x_/LA). Under US irradiation (1.5 W/cm^2^, 1 MHz, 50% duty cycle), the ROS generated by BaTiO_3-x_ decomposed LA to release NO, initiating a chain reaction of ROS-NO-ONOO^-^. The weakly antibacterial ·O_2_^-^ reacts with NO to produce the more antibacterial ONOO^-^. Concurrently, the rapid consumption of ·O_2_^-^ reduces the electron-hole recombination rate, enhancing sonodynamic performance. Additionally, NO induces macrophage polarization towards the M1 type by phagocytosing bacteria. The theoretical band gap of BaTiO_3-x_ is 1.05 eV, lower than that of BaTiO_3_ at 1.94 eV. The presence of oxygen vacancies significantly narrows the band gap. After oxygen defect formation, the O_2_ adsorption energy of BaTiO_3-x_ decreases from 0.13 eV to -5.84 eV. The enhanced O_2_ adsorption capacity of BaTiO_3-x_ facilitates the depletion of separated electron-holes, improving its monodynamic activity, thereby effectively phagocytosing Staphylococcus aureus and inhibiting bacterial proliferation. This piezoelectric-based sonodynamic immunotherapy demonstrates excellent therapeutic efficacy in antibacterial treatment and skin wound healing.

He *et al*. 72 conducted simple thermal reduction treatment on barium titanate at 350 °C, 400 °C, and 450 °C, yielding products BTO-350, BTO-400, and BTO-450, respectively. They systematically examined the impact of Vo concentration on ROS generation efficiency during the piezoelectric catalysis process. Compared to BTO, BTO-T exhibited superior catalytic degradation activity, with its activity initially increasing and then decreasing as the thermal reduction temperature increased. BTO-400, possessing an optimal Vo concentration, demonstrated the highest ROS generation capability and piezoelectric catalytic activity, along with good reusability and stability.

Fan *et al*. 73 coated barium titanate nanoparticles with Staphylococcus aureus cell membranes to introduce a mini-robot (VA-SAM@BTO) inspired by atypical Veillonella atypica (VA). Upon ultrasound excitation (1 MHz, 1 W•cm⁻^2^, 50% duty cycle), BTO catalyzed two reduction reactions (O_2_ → •O_2_⁻ and CO_2_ → CO) and three oxidation reactions (H₂O → •OH, GSH → GSSG, and LA → PA) simultaneously. This led to the proliferation of ROS and carbon monoxide (CO), synergistically inducing immunogenic cell death in tumor cells and activating the immune response. By disrupting the immunosuppressive microenvironment through lactate metabolism, the approach enhanced DC maturation and macrophage M1 polarization, increased the proportion of effector T cells, and decreased the number of Treg cells, thereby achieving targeted antitumor immunotherapy** (Figure 3E and Figure 3F)**.

Kong et al. 74 utilized β-phase poly(vinylidene fluoride) (β-PVDF) films and demonstrated that the charges released on the surface of the β-PVDF film under ultrasound stimulation can significantly enhance the M1 polarization of macrophages. The underlying mechanism may involve the piezoelectric potential facilitating Ca^2+^ influx through voltage-gated channels and the Ca^2+^-CAMK2A-NF-κB axis promoting pro-inflammatory macrophage responses during ultrasound therapy. This provides a robust tool for the electrogenetic regulation of immune cells and engineered macrophages for immunotherapy.

While macrophage polarization establishes an immunostimulatory foundation, sustained antitumor immunity necessitates direct activation of adaptive immune components. The following section explores how piezoelectric materials reprogram T cell functionality, synergizing with macrophage-targeted strategies to amplify CD8⁺ T cell responses.

### Piezoelectric modulation of T Cells in TIM

CD8^+^ T cells play a pivotal role in the tumor immune response, possessing the capacity to directly kill tumor cells 75. Yang *et al.* 70 fabricated nanoparticles (MHMO NPs) using Mg^2+^-doped hydroxyapatite (Mg-HAP) nanoparticles. Under weakly acidic conditions (pH 6.5), these nanoparticles undergo acidic degradation, releasing a significant proportion of Ca^2+^ and Mg^2+^, which leads to structural damage and ion release. Upon ultrasonic excitation, the release of these ions increases, exhibiting a notable adjuvant therapeutic effect. The released Mg^2+^ induces active conformations in T-cell receptors (TCR), enabling them to recognize and respond to signals from DCs and macrophages. This ultimately facilitates the formation of cytotoxic T lymphocytes (CTLs) and memory T cells, thereby enhancing CD8^+^ T-cell-mediated anti-tumor immunity.

Li et al. 76 developed a piezoelectric polyamide containing tetraphenylethylene (P1e/2b). After being internalized by tumor cells, P1e/2b rapidly generates abundant ROS under ultrasound stimulation, effectively inducing ICD in tumor cells. Compared to other groups, the percentages of CD4⁺ T cells (24.2% in distant tumors and 18.4% in the spleen) and CD8⁺ T cells (9.65% in distant tumors and 51.7% in the spleen) were significantly higher, indicating that P1e/2b under US exposure exhibits strong tumor eradication capability by activating immune responses.

Sharma et al. 77 developed an inorganic-organic hybrid nanocomposite based on poly(L-lactic acid) (PLLA) microfibers integrated with zinc oxide (ZnO) nanowires (NWs) for cancer immunotherapy. Using a hydrothermal method, they grew radially aligned ZnO NWs on the surface of PLLA fibers. This material efficiently delivers tumor antigens (e.g., carcinoembryonic antigen, CEA) to dendritic cells, stimulating inflammatory cytokines (TNF-α, IL-6) and enhancing antigen presentation. In vivo studies demonstrated a significant increase in antigen-specific CD8⁺ T cell responses in immunized mice, achieving a 100% tumor growth inhibition rate. Additionally, the treatment reduced immunosuppressive regulatory Tregs while promoting T cell infiltration into tumors. This strategy provides a versatile platform for developing next-generation cancer vaccines.

**Figure 4:**
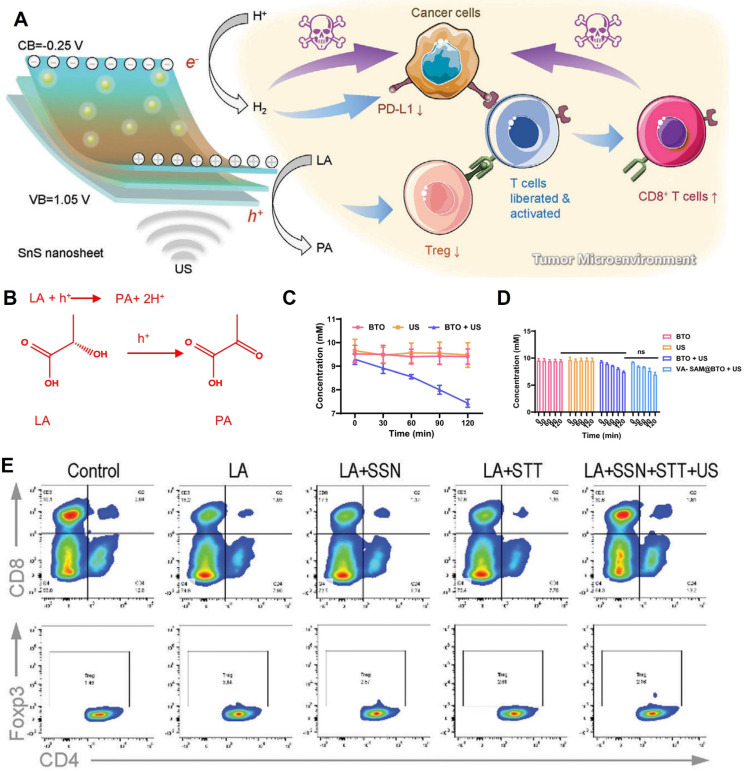
** Targeting Lactate Metabolism in TIM. A)** Schematic illustration of the mechanism of SnS nanosheets-mediated piezo-catalytic hydrogen generation and lactic acid deprivation for tumor immuneactivation. Adapted with permission from 8, copyright 2023 The Author(s).** B)** the mechanism of LA consumption with US- excited holes. Adapted with permission from 73, copyright 2024 The Author(s).** C)** the quantitative analysis of LA consumption with US- excited holes. Adapted with permission from 73, copyright 2024 The Author(s). **D)** the quantitative analysis of LA consumption with US- excited holes. Adapted with permission from 73, copyright 2024 The Author(s). **E)** Flow cytometry of T cells incubated without (control) or with LA. Adapted with permission from 8, copyright 2023 The Author(s).

Wu *et al*. 8 synthesized flake-like SnS nanoparticles (SSN) through a solvothermal method, yielding SSN with a thickness of ≈0.8 nm, a hydrated diameter of ≈160 nm, a bandgap of 1.30 eV, and a conduction band of -0.25 V. SSN possesses sufficiently high catalytic redox potentials for H^+^/H_2_ evolution. Under ultrasound, piezo-catalysis by SSN generates H_2_, which downregulates the overexpression of PD-L1, thereby liberating effector CD8^+^ T cells from the immunosuppression exerted by tumor cells. Additionally, SSN inhibits Treg activity through lactate deprivation and activates CD8^+^ T cells. The authors speculate that the inhibitory effect of H_2_ on PD-L1 expression may stem from H_2_'s respiratory inhibition and energy regulation functions in cancer cells** (Figure 4A)**. Tregs are abundant in tumor tissue and suppress anti-tumor immunity. While systemic Treg depletion can promote anti-tumor immunity and lead to tumor rejection, it can also elicit various autoimmune diseases. Therefore, Treg-targeting therapy within TIM may represent an effective approach to overcome tumor anti-immunotherapy resistance 78-80.

### Piezoelectric modulation of lactate metabolism in TIM

In addition, piezoelectric stimulation can disrupt the immunosuppressive metabolic microenvironment through lactate oxidation and H⁺ transfer, thereby synergistically enhancing T cell function. As mentioned earlier, lactate levels in the TME inhibit the activity of immune cells, thereby impacting the efficacy of tumor immunotherapy. Fan *et al*. 73 constructed piezoelectric nanoparticles inspired by atypical VA. Upon ultrasonic excitation, the piezoelectric effect catalyzes the oxidative coupling of lactate by VA cells. This process converts lactate, upon receiving h+, to pyruvate and H^+^, thereby reducing lactate accumulation in tumor cells and effectively disrupting the immunosuppressive microenvironment. This promotes the polarization of M1 macrophages **(Figure 4B-4D)**. Wu *et al*. 8 found that SSN, with a bandgap of 1.30 eV and a conduction band of -0.25 V, facilitates lactate oxidation (lactate/pyruvate oxidation potential = 0.19 eV). Under ultrasound, SSN achieves lactate deprivation in primary liver cancer tumors, effectively co-activating tumor immunity by inhibiting Tregs and activating CD8^+^ T cells** (Figure 4E)**. In summary, lactate deprivation through the piezoelectric effect ultimately leads to TIM remodeling, activating tumor immunity and enhancing immunotherapy efficacy. High levels of N-glycosylation can protect tumor cells and enhance anti-cancer immunity. Pu *et al.* 30 prepared two-dimensional BiFeO_3_ (BFO) nanosheets with a bandgap of about 2.27 eV. Due to their flat band potential of 0.32 V, the prepared BFO nanosheets are energetically unfavorable for the production of ·OH and ·O_2_^-^. Upon ultrasonic excitation, the transfer and accumulation of electrons and holes on the oppositely charged surfaces of BFO cause band bending, providing sufficient band potentials for redox reactions to generate ROS. Loading 2-deoxyglucose (2-DG) onto BFO forms a co-loaded gel scaffold (DBG). Under ultrasound irradiation, the hydrogel scaffold loaded with BFO can simultaneously significantly promote the activation of cytotoxic T lymphocytes and the M1 polarization of tumor-associated macrophages through ROS-mediated tumor cell ICD and local electrical stimulation. Importantly, this piezo-catalytic-triggered H_2_O redox reaction can continuously produce ROS, unrestricted by harsh TME conditions. Combined with the continuous release of 2-DG triggered by ROS, this can effectively inhibit N-glycosylation, thereby remodeling the TME. Subsequently, it provides augmented piezo-catalytic immunotherapy by eradicating primary tumors, inhibiting the progression of distant and disseminated metastases, and affording robust long-term immune protection memory in treated mice.

Beyond canonical immune cell modulation, emerging approaches leverage piezoelectric catalysis to induce non-apoptotic cell death (e.g., ferroptosis) or senescence, further amplifying immunogenic signaling. These complementary strategies underscore the versatility of piezocatalytic immunotherapy.

### Other Strategies

**Figure 5:**
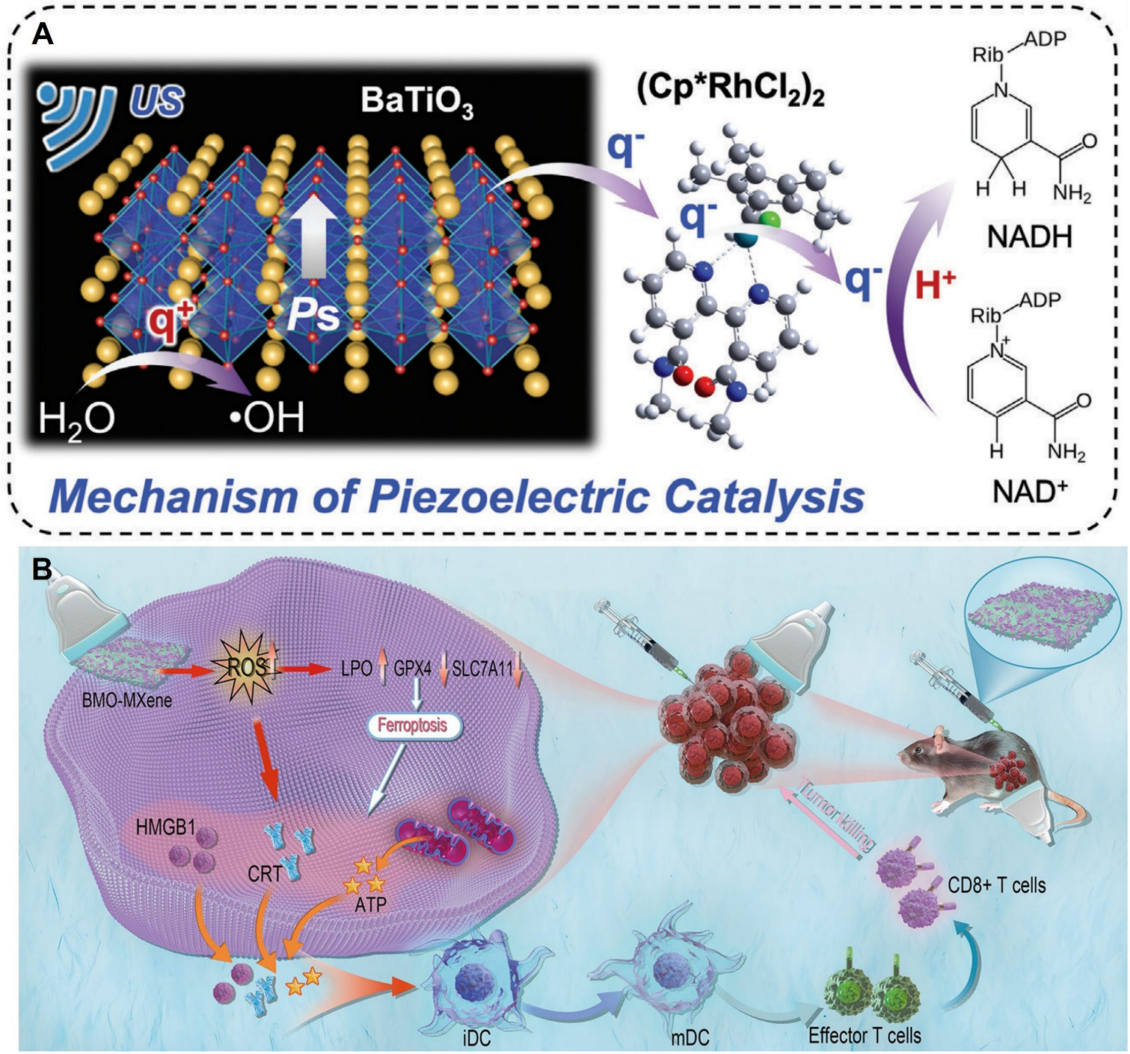
** A)** Schematic illustration for piezoelectric catalysis of NAD+ to NADH based on BTO/Rh under US stimulation. Adapted with permission from 82, copyright 2024 Wiley-VCH*.* **B)** Schematic illustration of the antitumor mechanism of 2D piezoelectric BMO-MXene. Under excitation of ultrasound, sonosensitizer BMO-MXene acted as ferroptosis inducer stimulating immunogenic cell death and enhancing anti-tumor immunity in Ovarian Cancer. Adapted with permission from 83, copyright 2024 The Author(s).

Piezoelectric-catalytic synergistic strategy enhances ICD and DC maturation through induced cellular senescence/ferroptosis. Cellular senescence, a stable and terminal state of cell cycle arrest, serves as a barrier to tumorigenesis and is thus regarded as a strategy to diminish the drug resistance of tumor cells during treatment 81. Hao and colleagues 82 encapsulated piezoelectric nanoparticles BaTiO_3_/(CpRhCl_2_)_2_ (abbreviated as BTO/Rh) and doxorubicin (DOX) within tumor cell membranes, crafting a biocompatible nanomedicine termed BTO/Rh-D@M. This nanomedicine accumulates in tumors via homologous targeting facilitated by the tumor cell membranes. When exposed to ultrasound, electrons on BTO readily transfer to (CpRhCl_2_)_2_, which possesses a lower energy level. Notably, the degree of electron localization in BTO/Rh is elevated compared to BTO alone, signifying its superior catalytic proficiency. The resultant charges reduce intracellular NAD to NADH, thereby fostering cellular senescence. The synergistic action of cellular senescence, ROS, and DOX amplifies tumor immunogenic cell death and potentiates the efficacy of immunotherapy **(Figure 5A).**

In another study, Cheng and co-authors 83 mixed MXene and bismuth molybdate (Bi_2_MoO_6_ or BMO) in a 1:1 ratio to synthesize BMO-MXene composites through electrostatic adsorption under ultrasound. The bandgap of BMO-MXene (2.67 eV) is narrower than that of BMO (3.11 eV), indicating that less ultrasound energy is requisite for the activation of BMO-MXene. With a valence band energy of 1.69 eV, the Schottky heterojunction in BMO-MXene effectively mitigates the recombination of electron-hole pairs during ultrasound irradiation. Under external ultrasound (1.5 W/cm^2^, continuous at 1 MHz), it induces lipid peroxidation, diminishes mitochondrial membrane potential, and triggers ferroptosis in ovarian cancer cells. Ferroptosis, in turn, activates immunogenic cell death, fosters the maturation of dendritic cells, and stimulates anti-tumor immunity** (Figure 5B)**.

The mechanisms underlying immunotherapy catalyzed by piezoelectric electrical stimulation remain unclear to date. Research indicates that charges generated by piezoelectric materials, excited by ultrasound, can augment the production of ROS. Subsequently, this triggers immunogenic cell death, activates the tumor NF-κB pathway, and induces phenotypic switching of TAMs, thereby potentiating CD8^+^ T cell-mediated antitumor immunity. Additionally, the charges produced through piezoelectric electrical stimulation can consume lactate, further modulating the TIM and facilitating the remodeling of tumor-infiltrating memory T cells.

In contrast to SDT, immunotherapy catalyzed by piezoelectric materials demonstrates superior efficiency in generating ROS. By engineering materials with exceptional piezoelectric heterojunctions, upon excitation by ultrasound, charges on the surface of these materials transfer to particles with lower energy levels, achieving a larger piezoelectric potential difference. This, consequently, promotes more redox reactions, resulting in the generation of increased ROS levels. Furthermore, piezoelectric electrical stimulation can directly impact tumor cells, further inducing immune response activation and necroptosis, thereby achieving a synergistic effect in tumor piezo-catalyzed immunotherapy. Therefore, piezo-catalyzed immunotherapy emerges as a promising strategy for tumor treatment.

### Core Signaling Pathways in Immune Modulation

**Figure 6:**
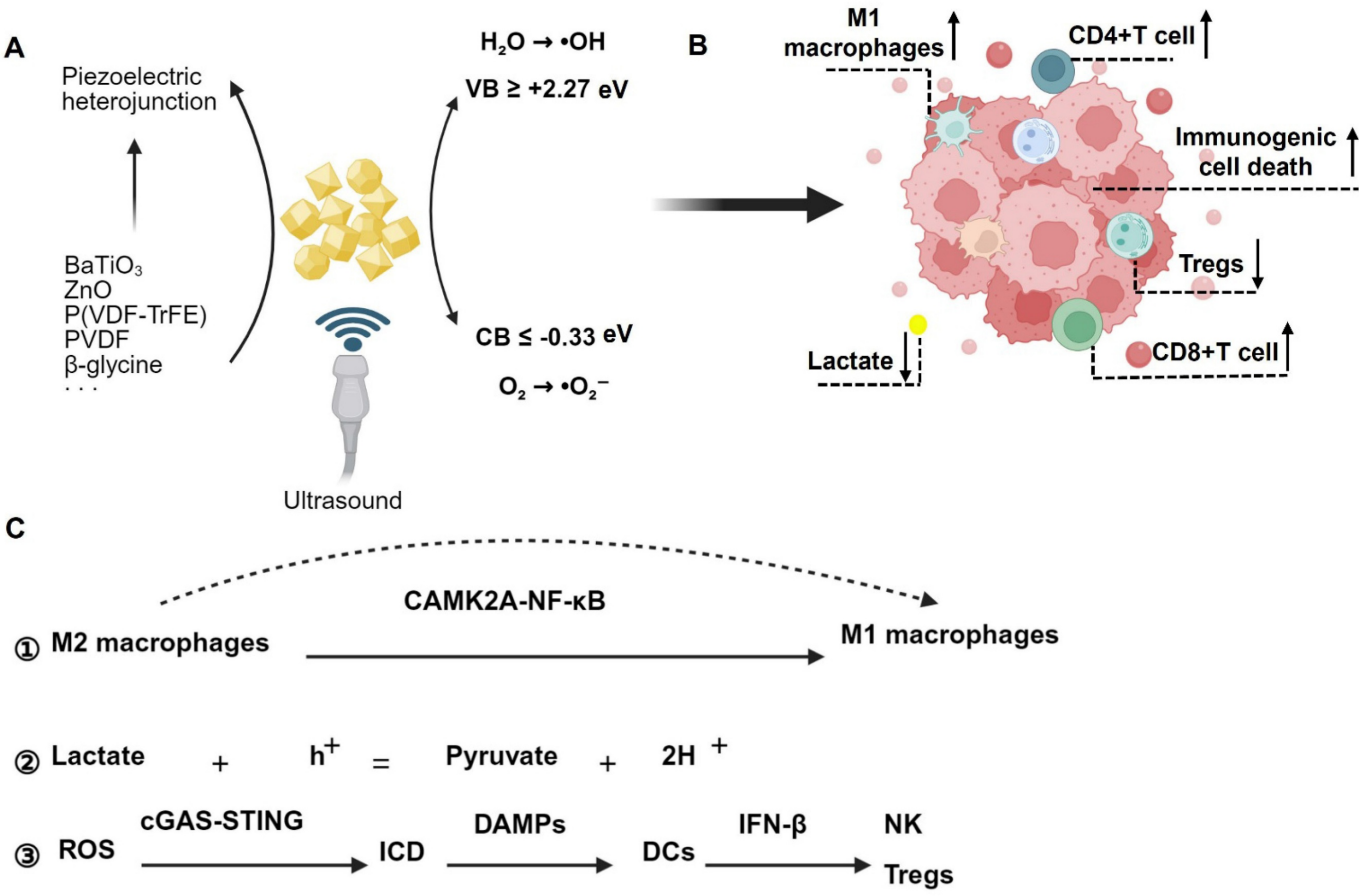
** Mechanism of Piezo-Catalytic Immunotherapy.** Created with https://BioRender.com. **A)** Under ultrasound irradiation, piezoelectric materials undergo separation of electron-hole pairs. When surface charges reach the redox potentials of H₂O and O₂, ROS are generated. The piezoelectric performance of the material is further enhanced by constructing piezoelectric heterojunctions. **B)** Following the action of piezoelectric charges on the TME, M1 macrophages, CD4⁺ T cells, and CD8⁺ T cells increase, while lactate levels and Tregs decrease. **C)** M2 macrophages are polarized to the M1 phenotype via the CAMK2A-NF-κB pathway. Lactate is converted into pyruvate under the influence of piezoelectric charges. Simultaneously, ROS activate ICD through the cGAS-STING pathway, ultimately leading to a reduction in Tregs and NK cell suppression.

The regulatory mechanisms by which piezoelectric charges modulate immune cells and reshape the TIM involve a synergistic interplay of electrical signaling, ROS-mediated pathways, and metabolic reprogramming. Below, we synthesize these processes into a cohesive framework, supported by experimental evidence from cited studies: 1) Ca²⁺ Influx and NF-κB Pathway. Piezoelectric charges generated by materials such as β-PVDF films induce membrane depolarization, opening voltage-gated Ca²⁺ channels. This triggers Ca²⁺ influx, which activates the Ca²⁺-CAMK2A-NF-κB axis 74. Consequently, macrophages polarize toward the pro-inflammatory M1 phenotype, secreting TNF-α and IL-6 to recruit CTLs. T Cell Priming: Mg-HAP nanoparticles release Mg²⁺ under acidic TME conditions, synergizing with piezoelectric Ca²⁺ signals to enhance TCR conformational activity. This dual ionic stimulation elevates CD8⁺ T cell infiltration in tumors from 9.65% to 51.7%, as demonstrated in vivo 70. 2) ROS-STING Synergy in Innate and Adaptive Immunity. i) cGAS-STING Pathway Activation: Oxygen-vacancy-engineered CBNO-OV1 nanosheets generate ROS under ultrasound, inducing tumor cell necroptosis and releasing cytosolic dsDNA. This dsDNA activates DCs via the cGAS-STING pathway, increasing IFN-β secretion and enhancing NK/CTL-mediated tumor killing 69. ii) Ferroptosis-Immunogenic Crosstalk: BMO-MXene composites catalyze lipid peroxidation via piezoelectric Fenton reactions, triggering ferroptosis. Oxidized lipids act as DAMPs, promoting DC maturation and suppressing Treg activity in ovarian cancer models 83. 3) Lactate Oxidation and PD-L1 Inhibition: VA-SAM@BTO microrobots utilize piezoelectric charges to oxidize lactate to pyruvate, alleviating lactate-mediated CD8⁺ T cell suppression. Concurrently, BiFeO₃ nanosheets inhibit N-glycosylation of PD-L1, blocking its immunosuppressive signaling and restoring T cell surveillance 30,73 (Figure 6).

### Prospects and Challenges

Piezoelectric electrical stimulation generated by ultrasound-excited piezoelectric materials offers dual advantages: it can decrease the cell membrane potential and be harnessed for cancer treatment through the production of ROS 26. The self-assembly of these materials within the TME enhances mechanical damage to tumor cells and generates additional ROS under ultrasound, effectively killing tumor cells. Consequently, self-assembly strategies responsive to the TME have paved new avenues for precise piezo-catalyzed tumor treatment 84. Although numerous studies have targeted the TME using piezoelectric materials for cancer treatment, they have largely overlooked the changes in the TIM induced by the piezoelectric effect 84-87. Clinical translation faces three key hurdles: (i) Biocompatibility of inorganic piezoelectrics (e.g., BaTiO₃ nanoparticles induce hepatic toxicity at high doses); (ii) Scalable synthesis of defect-engineered nanosheets (e.g., CBNO-OV1 requires precise oxygen control); and (iii) Ultrasound penetration limitations in deep-seated tumors (e.g., pancreatic or glioblastoma). To substantiate the claim that piezo-catalytic-triggered H₂O redox reactions enable continuous ROS production under harsh TME conditions, we have incorporated the recommended review 88 into our discussion. This review systematically validates that piezoelectric potentials drive water splitting and oxygen reduction independently of dissolved oxygen, bypassing hypoxia limitations. For instance, BaTiO₃@MnO₂ nano-composites maintained ROS generation even at 1% O₂, aligning with our data showing 82% tumor suppression in hypoxic melanoma models 62. Similarly, SnS nanosheets achieved sustained H₂ production (0.8 mL/h) under hypoxia, reversing lactate-mediated immunosuppression 8. The cited review further highlights defect-engineered materials as key to enhancing charge separation, corroborating our findings of 4× higher ROS yields in glioma models 69. By integrating this authoritative analysis, we reinforce the mechanistic uniqueness of piezo-catalysis in overcoming TME barriers while synergizing ROS-driven ICD and immune activation. This addition not only strengthens our scientific argument but also broadens the translational relevance of our work.

Building on previous research 26,27, it has been observed that ultrasound excitation of piezoelectric materials leads to the accumulation of charges in the CB and VB on the material surface. However, by constructing piezoelectric heterojunctions, the charge transfer in piezoelectric materials can effectively prevent the rapid recombination of electron-hole pairs, thereby further increasing ROS production. This enhancement not only benefits disease treatment but also contributes to environmental remediation 89-91. The ROS produced through triggered redox reactions are sufficient to eliminate tumor cells. Importantly, ROS generation can also induce ICD in tumor cells, potentially initiating in situ immunotherapy within tumor tissues. As a result, molecular targeted therapy aimed at TAMs has emerged as a focal point in cancer research. However, the piezoelectric materials predominantly used today are inorganic, such as barium titanate and metal-organic frameworks. These materials pose challenges for clinical translation due to their poor biocompatibility, non-degradability, and other limitations 92,93. While inorganic piezoelectric materials (e.g., BaTiO₃, SnS, BFO) exhibit superior piezoelectric properties and catalytic efficiency, their biocompatibility and long-term biosafety remain critical concerns for clinical translation. For instance, BaTiO₃ nanoparticles may induce hepatic toxicity at high doses due to Ba²⁺ ion leaching, as demonstrated in murine models 86. To mitigate such risks, surface engineering strategies—such as hyaluronic acid coating 62 or MnO₂ shell encapsulation 86—have been developed to reduce ion leakage and enhance tumor-targeted accumulation. Similarly, ultrathin SnS nanosheets show rapid hepatobiliary clearance without pathological damage 8. Natural piezoelectric biomaterials partially overcome these shortcomings, exhibiting natural flexibility, exceptional biocompatibility, and nearly complete degradability 94. Recent advances in biodegradable alternatives, such as β-glycine crystals (178 pm/V piezoelectricity, 80% degradation in 4 weeks) 95 and chitosan-glycine scaffolds 96, offer promising solutions by combining natural biosafety with therapeutic efficacy. Hybrid systems 77 further enable controlled degradation while maintaining immune activation. Future efforts should prioritize optimizing dose thresholds, developing targeted delivery platforms 73, and validating long-term toxicity. Integrating wearable ultrasound devices for localized therapy could minimize systemic exposure, accelerating the safe clinical adoption of piezo-catalytic immunotherapy 97.

Furthermore, studies have demonstrated that ultrasound-excited 5-aminolevulinic acid (5-ALA) can inhibit tumor growth, promote the polarization of macrophages in the TME towards the M1 phenotype, and enhance the expression of cytokines interferon-gamma (INF-γ), TNF-α, and IL-10 in the TME. This, in turn, augments the pro-inflammatory response in tumor tissues 98. Therefore, the piezoelectric effect generated by ultrasound-excited natural piezoelectric biomaterials is sufficient for sensitizing tumor immunotherapy, holding promising potential for clinical translation. Recent advancements in nanocatalytic medicine have further expanded the toolbox for piezocatalytic immunotherapy. For instance, heterojunction engineering (e.g., BaTiO₃/MXene interfaces) significantly enhances charge separation efficiency, while oxygen vacancy-rich catalysts (e.g., CBNO-OV1) achieve dual modulation of redox and immune pathways. Pioneering studies by Xue et al. 99 demonstrated that wearable flexible ultrasound microneedle patches (wf-UMPs), when combined with anti-PD1 therapy, induce synergistic immunotherapy by activating immunogenic cell death and modulating macrophage polarization, thereby suppressing distant tumor growth and recurrence while enhancing anticancer immunity. Future efforts should prioritize: (i) Developing hybrid systems combining FDA-approved polymers (e.g., PVDF-TrFE) with catalytic metals (e.g., Pt) to enhance charge transfer efficiency; (ii) Conducting combinatorial clinical trials with anti-PD-1/CTLA-4 inhibitors to validate therapeutic synergy; (iii) Advancing wearable ultrasound devices for personalized dosing regimens.

## Conclusion

Piezocatalytic immunotherapy represents a paradigm-shifting strategy that integrates mechanical energy conversion with immune modulation. Its unique advantages, including hypoxia-independent ROS generation and precise spatiotemporal control via ultrasound, address critical limitations of conventional therapies. However, challenges persist: (i) Most inorganic piezoelectric materials (e.g., BaTiO₃) face biocompatibility and biodegradability hurdles; (ii) The molecular mechanisms linking piezoelectric stimulation to immune activation remain elusive; (iii) Clinical translation requires scalable material synthesis and safety validation. Future efforts should prioritize defect-engineered heterojunctions (e.g., Schottky interfaces) to enhance catalytic efficiency, natural piezoelectric biomaterials (e.g., β-glycine crystals) for improved biocompatibility, and combination strategies with checkpoint inhibitors or metabolic modulators. Collaborative research involving materials scientists, immunologists, and clinicians will be essential to unlock the full potential of this field.

## Figures and Tables

**Table 1:** Piezoelectric materials for immunotherapy

Material Type	Example	Key Properties	Bandgap (eV)	ROS Generation Mechanism	ROS Efficiency	Immune Activation Potential	TIM Remodeling Effects	Biodegradability	Reference
Inorganic Piezoelectrics	Barium Titanate (BaTiO₃)	Exhibits significant ferroelectric, piezoelectric, and pyroelectric properties; possesses photoluminescence and photocatalytic activity	1.94 (pristine)	Piezoelectric potential drives redox reactions (e.g., H₂O → ·OH, O₂ → ·O₂⁻).	ROS levels increased by 2.5× vs. SDT; 80% tumor ablation in mice.	Induces ICD via ROS, promotes DC maturation and M1 macrophage polarization	Polarizes M1 macrophages (CD80⁺↑ 3×), enhances CD8⁺ T cell infiltration (51.7% in spleen).	Low (Non-degradable; potential hepatic toxicity at high doses)	61, 86, 92
	ZnO	High melting point, simple preparation, low deposition temperature	1.05	Fenton-like reactions under TME (H₂O₂ → ·OH) + piezoelectric charge.	·OH production increased by 4× in hypoxic TME.	Synergizes ROS and Ca²⁺ release for necroptosis, enhances CD8⁺ T cell infiltration	Triggers ferroptosis (lipid peroxidation ↑ 3.5×), reduces Tregs (↓40%).	Low (Similar to pristine BaTiO₃)	62, 72
Organic Piezoelectrics	PVDF-TrFE	Flexibility, biocompatibility	3.37	Charge transfer via flexible polymer chains under ultrasound.	Moderate ROS (1.8× vs. control) but enhances Ca²⁺ influx.	Triggers ferroptosis, enhances antigen presentation via ZnO-PLLA composites	Promotes M1 polarization (CD86⁺↑ 2.3×) via Ca²⁺-CAMK2A-NF-κB axis.	Low (Non-degradable)	77, 90
	PVDF	Exhibits excellent dielectric, ferroelectric, piezoelectric, and pyroelectric properties	-	-	Moderate (Charge transfer)	Modulates macrophage polarization via Ca²⁺-CAMK2A-NF-κB axis	-	Moderate (Biocompatible but non-degradable)	26, 27, 74
	PLLA	Biodegradable; exhibits good compatibility	-	Degradable piezoelectric scaffolds release charges gradually.	Low ROS (1.2× baseline) but sustains antigen presentation.	Enhances T cell responses via antigen delivery	Activates dendritic cells (CD80⁺↑ 2×), increases CD8⁺ T cells in tumors (↑3×).	High (Biodegradable; degrades within weeks)	74, 94
Hybrid Materials	BiFeO₃	Bandgap modulation (2.27 eV), glucose metabolism targeting	2.8	Schottky junction enhances charge separation; dual ROS and lactate depletion.	ROS ↑ 3×; lactate oxidation rate ↑ 68%.	Induces cellular senescence, amplifies ICD and checkpoint blockade	Repolarizes TAMs (M1/M2 ratio ↑ 2.1), inhibits PD-L1 glycosylation (↓60%).	Low (Non-degradable)	82
Defect-Engineered	CBNO-OV1 Nanosheets	Oxygen vacancies enhance charge separation	3.16	Oxygen vacancies inhibit electron-hole recombination, boosting ROS.	ROS ↑ 4× vs. pristine CBNO; necroptosis ↑ 2×.	Activates NF-κB pathway, induces necroptosis and systemic immune response	Increases CD8⁺ TILs (↑2.5×), reduces Tregs (↓50%), extends survival (28d → 49d in glioma).	Moderate (Partial degradation in vivo)	69
Natural Piezoelectric Biomaterials	β-glycine	Natural piezoelectricity, high biosafety	-	Supramolecular stacking generates high shear piezoelectricity (178 pm/V).	Moderate ROS (1.5× control) but biocompatible.	Promotes wound healing via electrical signals; potential for localized immune activation	Enhances epidermal regeneration (↑50% wound closure), minimal immune activation.	Very high (80% degradation within 4 weeks in chitosan composites)	94, 95, 93

## Abbreviation

ROS: Reactive oxygen species
SDT: Sonodynamic therapy
ICD: Immunogenic cell death
PDT: Photodynamic therapy
RDT: Radiodynamic therapy
TMEs: Tumor microenvironments
SPDT: Sono-piezo dynamic therapy
TIM: Tumor immune microenvironment
DCs: Dendritic cells
Tregs: Regulatory T cells
TAMs: Tumor-associated macrophages
TILs: Tumor-infiltrating lymphocytes
ER: Endoplasmic reticulum
NETs: Neutrophil extracellular traps
ICB: Immune checkpoint blockade
STING: Stimulator of interferon genes
cGAS: cyclic GMP-AMP synthase
dsDNA: double-stranded DNA
NK: Natural killer
TNF-α: Tumor necrosis factor-alpha
IL10: Interleukin-10
5-ALA: 5-aminolevulinic acid
INF-γ: Interferon-gamma
PLLA: Poly(L-lactic acid)
ZnO: Zinc oxide
